# Supplementary material for: Abundance modulates the ecosystem functional contributions of two sympatric Caribbean sea cucumbers
Source: PeerJ. 2023 Feb 16;11:e14823. doi: 10.7717/peerj.14823 (PMC9939023; doi:10.7717/peerj.14823)
Supplement: Supplemental Information 1 [file peerj-11-14823-s001.pdf]

Munger et al.

Abundance modulates the ecosystem functional contributions of two sympatric Caribbean sea cucumbers

Supplementary information

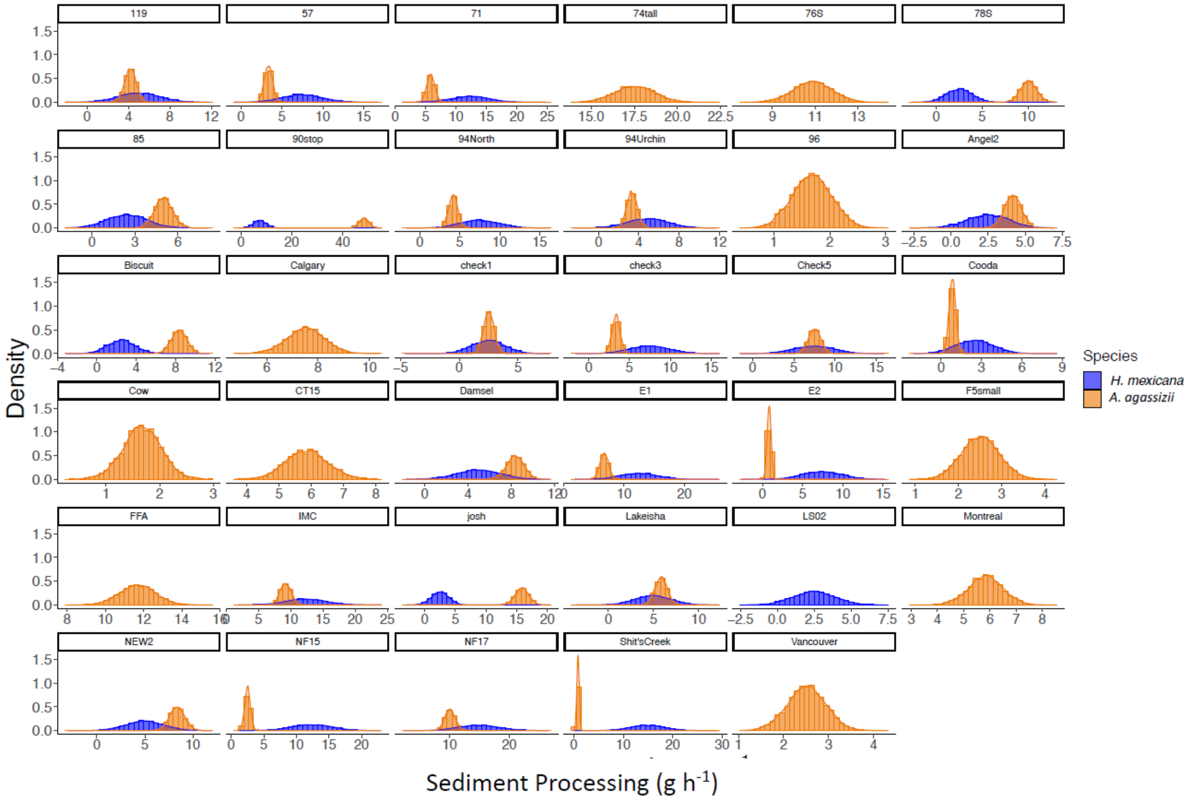

Figure S1. Bootstrap estimates of sediment processing (g h^{-1}) by *H. mexicana* (orange) and *A. agassizii* (blue) at 35 patch reef sites in Rock Sound, The Bahamas. Each data point is an estimate from one iteration, binned by the amount of sediment processing. Density is the proportion of data points in each bin, which sum to 1.

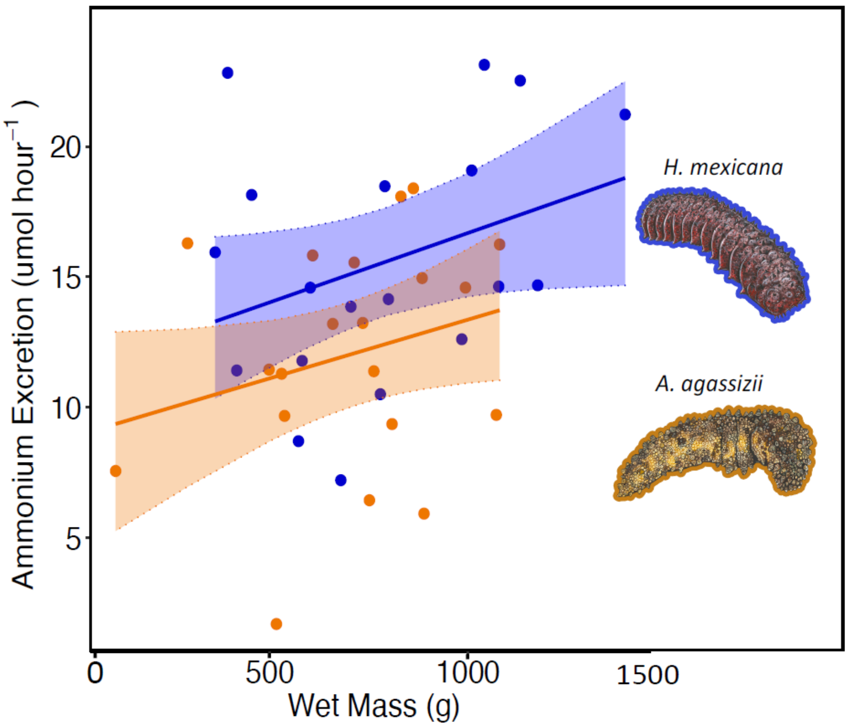

Figure S2. Relationship between ammonium excretion rate (µmol $NH4^+$ h^{-1}) and sea cucumber wet weight (g) for *H. mexicana* (orange line; y = 0.005x + 11.38, r^2 = 0.11, n = 19) and *A. agassizii* (blue line; y = 0.004x + 8.87, r^2 = 0.06, n = 20). The solid lines are the lines of best fit from the linear model; the shaded areas within the dashed lines are the 95% confidence intervals.

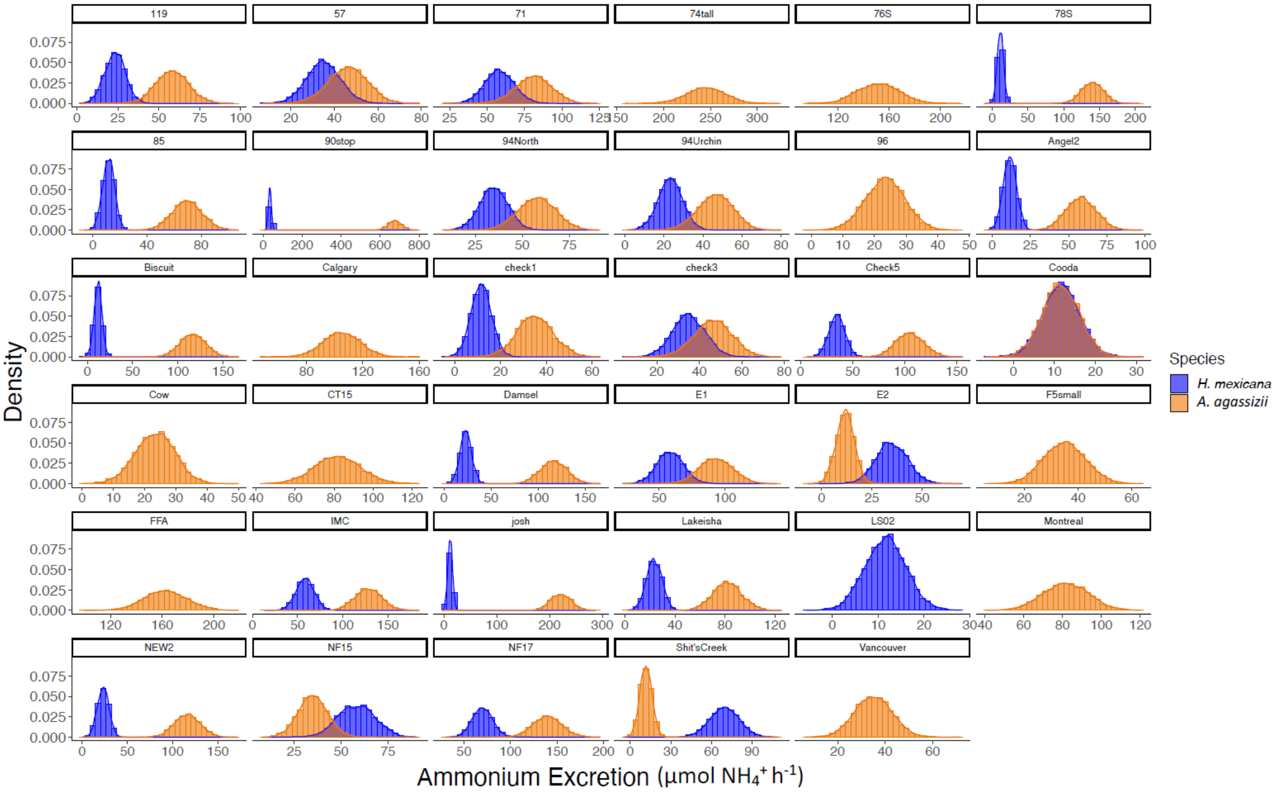

Figure S3. Bootstrap estimates of ammonium excretion rate (µmol $NH4^+$ h^{-1}) by *H. mexicana* (orange) and *A. agassizii* (blue) at 35 patch reef sites in Rock Sound, The Bahamas. Each data point is an estimate from one iteration, binned by the amount of ammonium excretion. Density is the proportion of data points in each bin, which sum to 1.
